# Sleep Quality and Insomnia Are Associated With Quality of Life in Functional Dyspepsia

**DOI:** 10.3389/fnins.2022.829916

**Published:** 2022-02-08

**Authors:** Fabien Wuestenberghs, Chloé Melchior, Charlotte Desprez, Anne-Marie Leroi, Marie Netchitailo, Guillaume Gourcerol

**Affiliations:** ^1^Department of Physiology, Rouen University Hospital, UNIROUEN, Normandy University, Rouen, France; ^2^INSERM Unit 1073, UNIROUEN, Normandy University, Rouen, France; ^3^Department of Gastroenterology and Hepatology, CHU UCL Namur, Université catholique de Louvain, Yvoir, Belgium; ^4^Department of Gastroenterology, Rouen University Hospital, UNIROUEN, Normandy University, Rouen, France; ^5^INSERM CIC-CRB 1404, Rouen University Hospital, UNIROUEN, Normandy University, Rouen, France; ^6^Department of Molecular and Clinical Medicine, Institute of Medicine, Sahlgrenska Academy, University of Gothenburg, Gothenburg, Sweden

**Keywords:** disorder of gut-brain interaction, functional dyspepsia, insomnia, quality of life, sleep disorders

## Abstract

**Background:**

Sleep disturbances are common in patients with functional dyspepsia. Our aim was to assess the relationship between subjective sleep and quality of life and to identify factors associated with impaired sleep in functional dyspepsia.

**Methods:**

One thousand two hundred and twenty patients referred for functional gastrointestinal disorders at a single tertiary care center between end 2017 and June 2019 were studied using a self-administered questionnaire. 355 patients with Rome IV-based functional dyspepsia were identified. Sleep was assessed using both the *Pittsburgh Sleep Quality Index* (PSQI) and the *Insomnia Severity Index* (ISI). The severity of dyspeptic symptoms was assessed using the *Total Symptom Score* (TSS). Quality of life was assessed by the *GastroIntestinal Quality of Life Index* (GIQLI). Anxiety and depression levels were evaluated using the *Hospital Anxiety and Depression* (HAD) scale.

**Key Results:**

Among the 355 patients with functional dyspepsia, 66 (18.6%) patients displayed normal sleep quality whereas 289 (81.4%) patients had altered sleep quality. Functional dyspepsia patients with sleep disturbances were older (48.1 ± 15.4 vs. 41.4 ± 16.0, *p* = 0.0009), had decreased quality of life (GIQLI: 75.3 ± 18.5 vs. 92.1 ± 15.4, *p* < 0.0001), greater severity of their symptoms (TSS: 18.9 ± 3.6 vs. 17.2 ± 3.9, *p* = 0.0007), and higher anxiety and depression scores (HADS: 17.7 ± 7.2 vs. 11.9 ± 5.1, *p* < 0.0001). A correlation was found between sleep quality and quality of life [*r* = −0.43 (95% CI: −0.51 to −0.34), *p* < 0.0001]. Independent factors predicting poor sleep quality were age [OR 1.03 (95% CI = 1.01–1.05), *p* = 0.006], depression level [OR 1.27 (95% CI = 1.16–1.39); *p* < 0.0001], and the severity of dyspeptic symptoms [OR 1.13 (95% CI = 1.04–1.22); *p* = 0.004].

**Conclusion and Inferences:**

A high prevalence of sleep disturbances was found in patients suffering from functional dyspepsia, with 81% of them having altered sleep quality and 61% having insomnia based on subjective assessment. Altered sleep quality and insomnia were associated with altered quality of life, higher severity of symptoms, and higher anxiety and depression scores in this disorder.

## Introduction

Functional dyspepsia (FD) is a disorder of gut-brain interaction (DGBI) affecting 7.2% of the general population in the Western world (Sperber et al., 2021a), defined by upper digestive complaints (epigastric pain and/or burning, early satiation, and/or postprandial fullness) lasting for more than 6 months with a negative workup according to the Rome committee (Stanghellini et al., 2016). In the general population, sleep disorders are frequent, are associated with upper and lower gastrointestinal symptoms and predict impaired quality of life (QoL) (Cremonini et al., 2009). They are known to be even more frequent in patients with FD compared to controls (Fass et al., 2000; Lu et al., 2005; Lacy et al., 2011; Futagami et al., 2013a; Shimpuku et al., 2014; Li et al., 2018), affecting over half of the patients in a recent meta-analysis (Andreev et al., 2021). However, sleep disturbance is not independently associated with functional dyspepsia in the general population (Vege et al., 2004). Sleep disorders are associated with symptoms severity and higher levels of anxiety and depression in FD patients (Lacy et al., 2011; Futagami et al., 2013a). Overlap with irritable bowel syndrome (IBS) with or without non-erosive reflux disease even worsens sleep quality in FD patients (Futagami et al., 2013b; Kim et al., 2018). Patients with FD symptoms also experience more daytime sleepiness (Morito et al., 2014; Wu et al., 2017). Impaired sleep impacts lives of patients with FD since it reduces work productivity and worsens economic loss in those patients (Matsuzaki et al., 2018). Finally, the presence of sleep disturbances is prognostic since lower baseline sleep disturbance was associated with improvement in dyspepsia scores over 3–6 months in a recent cohort study (Singh et al., 2021). However, the relationship between sleep disturbances and QoL has never been investigated in FD patients to our knowledge.

Our aim was to assess Rome IV-based FD patients about sleep quality and insomnia, to compare dyspeptic patients with normal vs. altered sleep quality, to assess the relationship between subjective sleep and QoL, and to identify factors associated with altered sleep quality and insomnia in functional dyspepsia.

## Materials and Methods

This study is based on retrospective analysis of prospectively acquired data from Rouen University Hospital. All patients had given written informed consent for data to be recorded and used for research, in accordance with the Declaration of Helsinki as revised in 2013 (World Medical Association [WMA], 2013). Approval was obtained from the French data protection authority (Commission Nationale de l’Informatique et des Libertés, CNIL No 817.917), in compliance with French legislation. Ethical approval for the analysis of the data was obtained (CERNI No E2021-45).

### Patients

We analyzed retrospectively all patients referred to our center for DGBI between December 2017 and June 2019 and who completed a self-administered questionnaire. The medical records were reviewed to exclude patients with organic illnesses (diabetes mellitus, coeliac disease, inflammatory bowel disease, history of surgery of the upper gastrointestinal tract, *H. pylori* gastritis). Duplicates were also excluded. Only patients fulfilling Rome IV criteria for functional dyspepsia were kept for analysis.

### Diagnostic Criteria and Assessment of Patients’ Characteristics

Functional dyspepsia and its subtypes, postprandial distress syndrome (PDS) and epigastric pain syndrome (EPS), as well as IBS were defined according to Rome IV criteria (Lacy et al., 2016; Stanghellini et al., 2016). Post-infectious origin to dyspeptic complaints was evaluated by asking for an episode of acute gastroenteritis during the month preceding the onset of symptoms (Parkman et al., 2010). Eight dyspeptic symptoms (postprandial fullness, abdominal pain, bloating, regurgitations, nausea, early satiety, belching, and vomiting) were collected using a 5-points Likert scale. Symptoms were graduated from 0 to 4 for the last 15 days, with 0 corresponding to absence and 4 to extremely severe. A *Total Symptom Score* (TSS) was used to evaluate dyspeptic symptoms severity (McCallum et al., 2005) and was calculated by adding the eight individual symptoms’ scores. The score ranges from 0 to 32, with higher scores reflecting more severe symptoms. Patients were also asked to report the duration of their dyspeptic symptoms (postprandial fullness, early satiation, and epigastric pain/burning). The *GastroIntestinal Quality of Life Index* (GIQLI), a validated score in French (Slim et al., 1999), was used to measure QoL. This score is composed of 36 items concerning symptoms, physical state, emotions, and social impact. The items are related to 5 dimensions: symptoms (19 items), physical function (7 items), emotional function (5 items), social integration (4 items), and the effect of any medical treatment (1 item). The global score ranges from 0 to 144; the higher the score is, the better is the QoL. Gastro-esophageal reflux (GER) symptoms were defined as feeling heartburn, regurgitations, or chest pain at least 2 times per week for more than 6 months. Anxiety and depression levels were also assessed, using the *Hospital Anxiety Depression Scale* (HADS) and its subscales (Zigmond and Snaith, 1983). Each subscale includes 7 items scored from 0 to 3: the higher the score, the higher the anxiety or depression levels. Patients were asked to self-report chronic fatigue. Gastric emptying was assessed by ^13^C-octanoic acid breath testing in a subgroup of patients, with the protocol of the study being explained in detail elsewhere (Wuestenberghs et al., 2019). A cut-off for half-emptying time of 166 min was used to discriminate patients with delayed gastric emptying (Bromer et al., 2002).

### Evaluation of Sleep

Sleep was assessed using the *Pittsburgh Sleep Quality Index* (PSQI) (Buysse et al., 1989) and the *Insomnia Severity Index* (ISI) (Bastien et al., 2001) questionnaires. The PSQI score ranges from 0 to 21; the higher the score, worse the sleep quality. Altered sleep quality was defined as a PSQI greater than five (89.6% sensitivity and 86.5% specificity) (Buysse et al., 1989). The ISI score is composed of 7 component scores, each weighted 0 to 3 points. Insomnia was defined as an ISI score greater or equal to ten (86.1% sensitivity and 87.7% specificity) (Morin et al., 2011). Different categories were individualized based on the results: no clinically significant insomnia for 0–7 points, subthreshold insomnia for 8–14 points, clinical insomnia (moderate severity) for 15–21 points, and clinical insomnia (severe) for 22–28 points.

### Study Design and Statistical Analysis

Consecutive patients with dyspeptic complaints were screened for FD according to Rome IV criteria. After descriptive statistics of the entire population, FD patients with normal and altered sleep quality according to the PSQI threshold were compared for their characteristics, QoL, and anxiety-depression scores. The same analyses were performed concerning insomnia, using the ISI results. Quantitative data were expressed by mean and standard deviation, and compared between subgroups using a non-gaussian unpaired *t*-test (Mann-Whitney test). Qualitative variables were for their part compared using Fisher’s exact test. Multiple groups comparisons were performed using Kruskal-Wallis test with Dunn’s multiple comparison post-test to compare all pairs of columns. The correlation between sleep quality expressed by the PSQI and quantitative variables (GIQLI, TSS, body mass index (BMI), HADS…) was determined using the Pearson correlation coefficient.

Finally, additional analyses included the search for factors associated with altered sleep quality and insomnia and analyzing the influence of gastric emptying in a subgroup of patients. A multivariate binary logistic regression model was used to identify factors associated with altered sleep quality and with insomnia, using backward Wald stepwise selection (entry probability 0.05 and removal probability 0.10). Adjusted odds ratios (OR) and their 95% confidence intervals (CI) were calculated. “altered sleep quality” and “insomnia” were used as the dependent variables, and age, BMI, duration of FD symptoms, gender, HADS-A and HADS-D subscales, overlap with IBS, smoking and TSS score were used as independent variables. Two-tailed *p*-values < 0.05 were considered statistically significant.

The data were extracted on spreadsheets using Microsoft Excel 365 for Windows (version 1903) and were analyzed using *GraphPad Prism* version 5.03 for Windows (G*raphPad* Software Inc., San Diego, CA, United States)^1^, except multivariate analysis which was performed using SPSS software version 20.0.0 (IBM Corp. Released 2013. IBM SPSS Statistics for Windows, Version 20.0. Armonk, NY: IBM Corp).

## Results

### Patient Characteristics

One thousand two hundred and twenty patients referred during the study period were screening for inclusion. After excluding duplicates (*n* = 114) and patients not fulfilling Rome IV criteria for functional dyspepsia (*n* = 751), a total of 355 (29.1%) patients were analyzed. Among them, 227 (63.9%) patients had PDS subtype, 41 (11.6%) EPS subtype, and 87 (24.5%) PDS + EPS overlap. A female predominance was present (76.6%). Mean age of the patients was 46.9 ± 15.7 years. Mean duration of FD symptoms was 6.4 ± 8.3 years. Overlaps with IBS and GER symptoms were present in 159 (44.8%) and 181 (51.0%) patients, respectively. Clinical and demographic characteristics of the overall population are presented in Table 1.

**TABLE 1:** Patient characteristics of the overall population.

	Patients (*n* = 355)
Median age, years (range)	47 (18–83)
Mean BMI, kg.m^–2^ (± SD)	24.1 (± 5.2)
Sex ratio Male/Female (% women)	0.3 (76.6)
Smoking, *n* (%)	60 (16.9)
IBS, *n* (%)	159 (44.8)
Any GER symptom, *n* (%)	181 (51.0)
Gastric half-emptying time, minutes (± SD)*	164.4 (± 59.0)
Delayed gastric emptying, *n* (%)*	31 (39.7)
Median duration of FD symptoms, months (range)	36 (7–720)
TSS, mean score (± SD)	18.6 (± 3.7)
GIQLI, mean score (± SD) and its domains: Symptoms, mean score (± SD) Physical function, mean score (± SD) Emotional function, mean score (± SD) Social function, mean score (± SD) Treatment, mean score (± SD)	78.4 (± 19.1) 44.4 (± 8.8) 11.4 (± 5.9) 10.0 (± 4.3) 9.4 (± 4.1) 3.2 (± 1.2)
HADS, mean score (± SD) HADS-A, mean subscale (± SD) HADS-D, mean subscale (± SD)	16.6 (± 7.2) 9.9 (± 4.2) 6.7 (± 4.1)

*BMI, body mass index; EPS, epigastric pain syndrome; FD, functional dyspepsia; GER, gastro-esophageal reflux; GIQLI, gastrointestinal quality of life index; HADS, Hospital Anxiety Depression Scale; HADS-A and HADS-D, HADS Anxiety and Depression subscales, respectively; IBS, Irritable Bowel Syndrome; n, number; PDS, postprandial distress syndrome; SD, standard deviation; TSS, Total Symptom Score.*

* Only available for a subgroup of 78 (22.0%) patients.

Altered sleep quality was present in 289 (81.4%) of the patients while insomnia was present in 215 (60.6%) of the patients. A total of 117 patients had taken at least one sleeping pill over the past month, whose 76 took sleep pills regularly. Chronic fatigue was self-reported by 139 patients (39.2%), with 92.8% of them presenting poor sleep quality compared to 74.1% of those without this complaint [OR 4.52 (95% CI = 2.22–9.20), *p* < 0.0001]. Detailed characteristics of subjective sleep in our population are presented in Table 2.

**TABLE 2:** Detailed characteristics of subjective sleep in the overall population.

	Patients (*n* = 355)
Global PSQI, mean score (± SD)	8.9 (± 3.8)
PSQI component scores: Subjective sleep quality, mean score (± SD) Sleep latency, mean score (± SD) Sleep duration, mean score (± SD) Habitual sleep efficiency, mean score (± SD) Sleep disturbances, mean score (± SD) Use of sleeping medication, mean score (± SD) Daytime dysfunction, mean score (± SD)	1.6 (± 0.7) 1.7 (± 1.0) 1.0 (± 0.8) 1.0 (± 1.1) 1.6 (± 0.6) 0.8 (± 1.2) 1.2 (± 0.9)
Altered sleep quality (PSQI > 5), *n* (%)	289 (81.4)
ISI, mean score (± SD)	11.7 (± 6.3)
Categories of insomnia: No clinically significant insomnia, *n* (%) Subthreshold insomnia, *n* (%) Clinical insomnia (moderate severity), *n* (%) Clinical insomnia (severe), *n* (%)	108 (30.4) 126 (35.5) 100 (28.2) 21 (5.9)
Insomnia (ISI ≥ 10), *n* (%)	215 (60.6)

*ISI, insomnia severity index; n, number; PSQI, Pittsburgh sleep quality index; SD, standard deviation.*

### Comparison Between Patients With and Without Sleep Quality Impairment

Functional dyspepsia patients with altered sleep quality were older, had impaired QoL, greater severity of their symptoms, and higher anxiety and depression score. On the contrary, BMI, gender, smoking, duration of FD symptoms, FD subtypes distribution, and overlap with IBS, the reporting of GER symptoms and gastric emptying rate were the same in both groups. Results are detailed in Table 3.

**TABLE 3:** Comparison of FD patients with normal vs. altered sleep quality.

	Normal sleep quality (*n* = 66)	Altered sleep quality (*n* = 289)	*p*-Value
Mean age, years (± SD)	41.4 (± 16.0)	48.1 (± 15.4)	0.0009
Mean BMI, kg.m^–2^ (± SD)	23.2 (± 4.4)	24.3 (± 5.3)	0.11
Sex ratio Male/Female (% women)	0.3 (77.3)	0.3 (76.5)	> 0.99
Smoking, *n* (%)	7 (10.6)	53 (18.3)	0.2
IBS, *n* (%)	32 (48.5)	127 (43.9)	0.6
Any reflux symptom, *n* (%)	33 (50.0)	148 (51.2)	0.9
Gastric half-emptying time, minutes (± SD)*	152.8 (± 37.9)	166.5 (± 61.8)	0.5
Delayed gastric emptying, *n* (%)*	4 (33.3)	27 (40.9)	0.8
Mean duration of FD symptoms, months (± SD)	65.6 (± 90.3)	78.6 (± 101.8)	0.3
TSS, mean score (± SD)	17.2 (± 3.9)	18.9 (± 3.6)	0.0007
GIQLI, mean score (± SD) GIQLI domains: Symptoms, mean score (± SD) Physical function, mean score (± SD) Emotional function, mean score (± SD) Social function, mean score (± SD) Treatment, mean score (± SD)	92.1 (± 15.4) 48.2 (± 8.1) 15.7 (± 5.1) 12.2 (± 3.6) 11.6 (± 3.4) 3.5 (± 1.0)	75.3 (± 18.5) 43.9 (± 8.7) 10.8 (± 5.8) 9.7 (± 4.3) 9.1 (± 4.1) 3.2 (± 1.2)	< 0.0001 0.004 < 0.0001 0.0003 0.0001 0.08
HADS, mean score (± SD) HADS-A, mean subscale (± SD) HADS-D, mean subscale (± SD)	11.9 (± 5.1) 7.8 (± 3.5) 4.1 (± 2.9)	17.7 (± 7.2) 10.4 (± 4.3) 7.3 (± 4.1)	< 0.0001 < 0.0001 < 0.0001
Global PSQI, mean score (± SD)	3.9 (± 1.0)	10.1 (± 3.3)	< 0.0001
Altered sleep quality (PSQI > 5), *n* (%)	0 (0.0)	289 (100.0)	< 0.0001
ISI, mean score (± SD)	4.5 (± 3.2)	13.4 (± 5.7)	< 0.0001
Insomnia (ISI ≥ 10), *n* (%)	5 (7.6)	210 (72.7)	< 0.0001

*BMI, body mass index; EPS, epigastric pain syndrome; FD, functional dyspepsia; GIQLI, gastrointestinal quality of life index; HADS, Hospital Anxiety Depression Scale, HADS-A and HADS-D, HADS Anxiety and Depression subscales, respectively; IBS, irritable bowel syndrome; ISI, insomnia severity index; n, number; PDS, postprandial distress syndrome; PSQI, Pittsburgh sleep quality index; SD, standard deviation; TSS, Total Symptom Score.*

* Only available for a subgroup of 78 (22.0%) patients.

**FIGURE 1:**
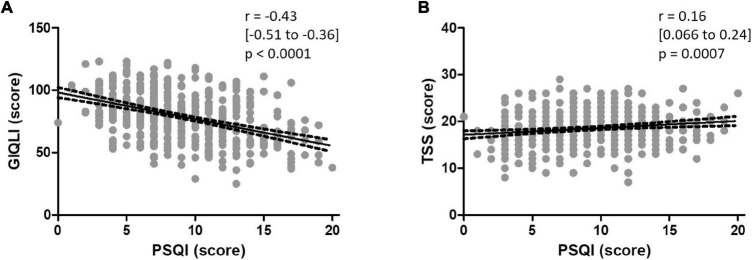
Correlation between the global PSQI and **(A)** the GIQLI and **(B)** the TSS, with the regression line best predicting the model and its 95% confidence band in both cases.

A correlation was found between sleep quality and QoL [*r* = −0.43 (95% CI: −0.51 to −0.36), *p* < 0.0001], each deterioration of one point in sleep quality assessed by the global PSQI being associated with a decrease of 0.43 point of QoL assessed by the GIQLI. Other parameters correlated with the sleep quality were age, BMI, anxiety and depression scales (HADS and its subscales), and symptoms severity assessed by the TSS (*r* = 0.21, 0.017, 0.37, and 0.16, respectively, all with *p* < 0.05), but not the duration of FD symptoms or the gastric half-emptying time (*r* = 0.29 and 0.20, respectively). Results are detailed in Supplementary Table 1 and depicted in Figure 1.

### Comparison Between Patients With and Without Insomnia

Functional dyspepsia patients with insomnia had impaired QoL, greater severity of their symptoms, and higher anxiety and depression scores. On the contrary, age, BMI, gender, smoking, and duration of FD symptoms, FD subtypes distribution, overlap with IBS and the reporting of GER symptoms were the same in both subgroups, except chest pain which was more frequent in those with insomnia. Results are detailed in Supplementary Table 2.

Pittsburgh sleep quality index and Insomnia severity index scores are highly correlated [*r* = −0.41 (95% CI: −0.50 to −0.32), *p* < 0.0001]. Only 1 (0.5%) patient displayed insomnia without altered sleep quality among all patients presenting insomnia, whereas 79 (27.3%) patients displayed altered sleep quality without insomnia among all patients presenting altered sleep quality.

### Factors Predicting Altered Sleep Quality and Insomnia in Functional Dyspepsia Patients

In multivariate analysis, predictive factors of poor sleep quality were age [OR 1.03 (95% CI = 1.01–1.05), *p* = 0.006], depression level assessed by the HADS-D subscale [OR 1.27 (95% CI = 1.16–1.39); *p* < 0.0001] and the severity of dyspeptic symptoms assessed by the TSS [OR 1.13 (95% CI = 1.04–1.22); *p* = 0.004], while other factors (BMI, duration of the symptoms, gender, HADS-A, overlap with IBS and smoking) were not. The model combining age, depression score and symptom severity score correctly predicted poor sleep quality in 82.5% of the cases. Results are shown in Table 4A.

**TABLE 4A:** Multivariate binary logistic regression model of factors predicting altered sleep quality in FD patients.

	*B*	*SE*	OR	95% CI	*p*-Value
Age	0.03	0.01	1.03	1.01–1.05	0.006
HADS-D	0.24	0.05	1.27	1.16–1.39	< 0.0001
TSS	0.12	0.04	1.13	1.04–1.22	0.004
Constant	−3.17	0.89	0.04		< 0.0001

*B, unstandardized regression coefficient; CI, confidence interval; HADS-D, Hospital Anxiety Depression Scale – Depression subscale; OR, odds ratio; SE, standard error; TSS, Total Symptom Score.*

Independent predictive factors of insomnia in our cohort were anxiety and depression levels assessed by the HADS-A and HADS-D subscales, respectively [OR 1.09 (95% CI = 1.02–1.16); *p* = 0.01 & OR 1.15 (95% CI = 1.07–1.24); *p* < 0.0001, respectively], while other factors (BMI, duration of the symptoms, gender, HADS-A, overlap with IBS, severity of dyspeptic symptoms and smoking) were not. Results are shown in Table 4B.

**TABLE 4B:** Multivariate binary logistic regression model of factors predicting insomnia in FD patients.

	*B*	*SE*	OR	95% CI	*p*-Value
HADS-A	0.08	0.03	1.09	1.02–1.16	0.01
HADS-D	0.14	0.04	1.15	1.07–1.24	< 0.0001
TSS	0.10	0.03	1.11	1.04–1.18	0.002
Constant	−3.11	0.67	0.05		< 0.0001

*B, unstandardized regression coefficient; CI, confidence interval; HADS-A, Hospital Anxiety Depression Scale – Anxiety subscale; HADS-D, Hospital Anxiety Depression Scale – Depression subscale; OR, odds ratio; SE, standard error; TSS, Total Symptom Score.*

## Discussion

We confirm the high prevalence of sleep disturbances in FD patients, with 81% of patients having altered sleep quality based on subjective assessment and 61% having insomnia. We show for the first time to our knowledge an association between altered sleep quality or insomnia and altered QoL in FD patients. We also confirm that altered sleep quality is associated with higher symptom severity and higher anxiety and depression scores in FD. Same results were obtained both using PSQI and ISI scores, considering that both scores were highly correlated. Our distribution pattern of FD subtypes is the same as reported in the literature (Aziz et al., 2018). We confirm that sleep quality is comparable among the 3 subtypes of FD (PDS, EPS, and PDS + EPS), as already shown by Yamawaki et al. (2014). The prevalence of IBS in our FD population was 45%, which corresponds to the pooled prevalence of 37% (95% CI = 30–45%) reported in a recent meta-analysis (Ford et al., 2010).

An increased prevalence of sleep disturbances compared to healthy subjects is found in most studies concerning FD patients (Fass et al., 2000; Lu et al., 2005; Lacy et al., 2011; Futagami et al., 2013a; Shimpuku et al., 2014; Li et al., 2018) but is not consensual for IBS (Kim et al., 2018; Koloski et al., 2021). However, the prevalence varies greatly among studies, depending on the definition used and the specific sleep disorder which is studied. Indeed, the International Classification of Sleep Disorders-third edition (ICSD-3) includes 83 disorders divided into seven main categories, with the major disorder being insomnia (Sateia, 2014). The prevalence of sleep disturbances in patients with FD was 53.2% (95% CI = 37.7–68.4) in a recent meta-analysis (Andreev et al., 2021), with significant heterogeneity between the results (*p* < 0.0001). Sleep disorders were more frequent in FD patients compared to healthy controls, with an odds ratio of 2.9 (95% CI = 2.5–3.3).

Patients suffering from FD have decreased QoL (El-Serag and Talley, 2003). Known risk factors for impaired health-related QoL in FD areanxiety and depression (Haag et al., 2008), increasing age (Hantoro et al., 2018), female gender (Talley et al., 2006), greater symptom severity (Haag et al., 2008), low-to-mid educational level (Hantoro et al., 2018), and somatization (Van Oudenhove et al., 2011), greater overlap with other disorders of gut-brain interaction (Sperber et al., 2021b), … but not gastric emptying (Talley et al., 2006; Haag et al., 2010). Interestingly, amitriptyline has been shown to modestly improve quality of sleep in dyspeptic patients (Herrick et al., 2018). Since we found a correlation between QoL and sleep quality in our cohort, sleep determinants should be considered among factors associated with QoL in this disorder. Furthermore, especially since altered sleep quality and/or insomnia are associated with altered QoL in FD patients, sleep could represent a target in future clinical trials in this disorder. Indeed, altered sleep quality has been shown to have a negative effect on the prognosis of FD (Yu et al., 2013; Chen et al., 2016; Singh et al., 2021). Recently, a non-randomized trial involving 20 patients suggested that adding sleep aids could improve gastrointestinal symptoms and QoL in FD patients (Nakamura et al., 2021).

Mechanisms underlying the association between FD and altered sleep are poorly understood but are probably complex. Knowing if sleep disturbances are responsible for the occurrence of FD or is a consequence of the disorder is debated (Maneerattanaporn and Chey, 2009). Interestingly, difficulty falling asleep and altered sleep associated with worsening symptoms were independent risk factors for dyspepsia in a community-based cohort (Gathaiya et al., 2009), together with somatization. It is logical to assume that digestive complaints could interact with sleep induction or continuation but altered sleep could also increase symptoms’ perception by patients. Indeed, sleep deprivation has been shown to decrease pain thresholds in healthy volunteers (Onen et al., 2001). Altered sleep could therefore exacerbate digestive complaints in these patients. This vicious circle could explain the association between altered sleep quality and higher symptoms severity that we found in our work. Independent factors predicting altered sleep quality in FD patients in our study were age, depression levels and symptoms severity. Gastro-esophageal reflux disease (GERD) is the most frequently reported factor involved in the pathogenesis of sleep alterations in FD. Indeed, sleep alterations tend to be aggravated by heartburn and regurgitation (Vakil et al., 2016), and an antisecretory drug being shown to improve sleep in FD patients (Futagami et al., 2013a). Furthermore, patients with GERD overlapping with FD have more altered sleep quality compared to those with GERD alone (Lei et al., 2019). Gastroesophageal reflux can awake patients or delay them falling asleep, but can also induce brief arousals leading to fragmented sleep (Lacy et al., 2011). We didn’t confirm a link between GERD and altered sleep in our study, probably because only GER symptoms were assessed and not GERD. Moreover, the pathophysiology of sleep disturbances has been shown to involve alterations in rapid eye movement sleep (Kumar et al., 1992) and the autonomic nervous system (Jarrett et al., 2008) in IBS; whether it is the same in FD remains to be determined. No relationship was found between sleep quality and gastric emptying rate in FD patients in our study, confirming the results of Futagami et al. (2013a). This is explained by the absence of effect of gastric emptying on esophageal acid exposure (Gourcerol et al., 2013). Age seems not to explain the association between FD and altered sleep since there is no association between age and sleep quality in the general population (Hinz et al., 2017). However, we found sleep disturbances to be associated with older age in our cohort. The presence of an overlap with other DGBI could also play a role since patients with overlapping FD-IBS have greater sleep quality impairment compared to those with FD alone (Kim et al., 2018). In addition, we did not find a correlation between the duration of FD symptoms and the quality of sleep. However, we found an association between sleep disturbances and greater severity of FD symptoms. Similarly, a Chinese study found that FD patients who had sleep disorders had a higher morbidity rate than those who suffered from IBS or functional constipation (Zhao et al., 2018). Sleep disorders were already shown to be more frequent in refractory FD compared to non-refractory disease (Jiang et al., 2015). Finally, other psychological factors, like anxiety, depression and somatization, could also participate in the induction of sleep disturbances. Interestingly, a discrepancy between objective and subjective assessments of sleep quality has been reported in IBS patients, suggesting altered sleep perception in this disorder (Elsenbruch et al., 1999).

Limitations of our work include that it is retrospective, monocentric, and from a tertiary center. Furthermore, we used a convenience cohort, which could not be representative of the entire FD population. GERD was not screened since no esophageal pH monitoring was performed; only GER symptoms are reported in our work. Use of sleep medication was not recorded and we did not have a control group composed of healthy volunteers to compare our results with. Gastric emptying was only assessed in a subgroup of patients, with likely a selection bias. Somatization was not assessed even if it could partly explain the association between FD and sleep dysfunction (Gathaiya et al., 2009). Another limitation is the use of self-reported sleep quality, using a questionnaire, instead of objective measures like polysomnography or actigraphy. Furthermore, the PSQI is known to be influenced by psychological factors, especially depression state (Buysse et al., 1989). Higher anxiety and depression levels in our population could therefore exacerbate erroneously the association between sleep disturbances and QoL.

## Conclusion

Most of the patients with FD experience sleep disturbances, with altered sleep quality and insomnia in 81 and 61% of them, respectively. Altered quality of sleep and/or insomnia are associated with altered QoL, higher severity of symptoms and higher anxiety and depression scores in this disorder. Older age, higher depression scores and severity of dyspeptic symptoms predict altered quality of sleep in FD patients.

## Data Availability Statement

The original contributions presented in the study are included in the article/Supplementary Material; further inquiries can be directed to the corresponding author.

## Ethics Statement

The studies involving human participants were reviewed and approved by CERNI (Comité d’Éthique de la Recherche Non-interventionnelle), Rouen University Hospital, Rouen, France. The patients/participants provided their written informed consent to participate in this study.

## Author Contributions

GG and FW were involved in acquisition of data, designed the study, contributed to analysis and interpretation of data, and drafted the manuscript. A-ML, CD, CM, GG, MN, and FW were provided critical revision of the manuscript for important intellectual content. All authors contributed to manuscript revision, read, and approved the submitted version.

## Conflict of Interest

The authors declare that the research was conducted in the absence of any commercial or financial relationships that could be construed as a potential conflict of interest.

## Publisher’s Note

All claims expressed in this article are solely those of the authors and do not necessarily represent those of their affiliated organizations, or those of the publisher, the editors and the reviewers. Any product that may be evaluated in this article, or claim that may be made by its manufacturer, is not guaranteed or endorsed by the publisher.

## Footnotes

1
www.graphpad.com

## Abbreviations

BMI: body mass index
CI: confidence interval
DGBI: disorders of gut-brain interaction
EPS: epigastric pain syndrome
FD: functional dyspepsia
GERD: gastroesophageal reflux disease
GI: gastrointestinal
GIQLI: gastrointestinal quality of life index
HAD: Hospital Anxiety and Depression
HADS: HAD scale
HADS-A: HADS anxiety subscale
HADS-D: HADS depression subscale
IBS: irritable bowel syndrome
ISI: insomnia severity index
OR: odds ratio
PDS: postprandial distress syndrome
PSQI: Pittsburgh Sleep Quality Index
QoL: quality of life
SD: standard deviation
TSS: Total Symptom Score.
