# Supplementary material for: Sleep Quality and Insomnia Are Associated With Quality of Life in Functional Dyspepsia
Source: Front Neurosci. 2022 Feb 8;16:829916. doi: 10.3389/fnins.2022.829916 (PMC8861511; doi:10.3389/fnins.2022.829916)
Supplement: Supplementary file 1 [file Table_1.DOCX]

Supplementary Material

**Supplementary table 1.** Correlations between the global PSQI and different quantitative parameters in our population using Pearson correlation coefficient.

|  | **R** | **95% CI** | **p-value** |
| --- | --- | --- | --- |
| ***Age*** | 0.21 | 0.11 to 0.30 | < 0.0001 |
| ***BMI*** | 0.017 | -0.087 to 0.12 | 0.017 |
| ***Duration of FD symptoms*** | 0.056 | -0.048 to 0.16 | 0.29 |
| ***GIQLI*** | -0.43 | -0.51 to -0.34 | < 0.0001 |
| ***HADS*** | 0.37 | 0.27 to 0.45 | < 0.0001 |
| ***HADS-A*** | 0.28 | 0.18 to 0.38 | < 0.0001 |
| ***HADS-D*** | 0.36 | 0.26 to 0.45 | < 0.0001 |
| ***ISI*** | -0.41 | -0.50 to -0.32 | < 0.0001 |
| ***T1/2*** | 0.20 | -0.021 to 0.41 | 0.076 |
| ***TSS*** | 0.16 | 0.060 to 0.26 | 0.0021 |
| **BMI: Body Mass Index, CI: Confidence Interval, FD: Functional Dyspepsia, GIQLI: GastroIntestinal Quality of Life Index, HADS: Hospital Anxiety Depression Scale, HADS-A and HADS-D: HADS Anxiety and Depression subscales respectively, IBS: Irritable Bowel Syndrome, PSQI: Pittsburgh Sleep Quality Index, TSS: Total Symptom Score, T1/2: gastric half-emptying time** | | | |

**Supplementary table 2.** Comparison of FD patients with insomnia vs. no insomnia

|  | **No insomnia**  **(n = 140)** | **Insomnia**  **(n = 215)** | **p-value** |
| --- | --- | --- | --- |
| ***Mean age, years (± SD)*** | 45.24 (± 16.98) | 47.92 (± 14.74) | 0.099 |
| ***Mean BMI, kg.m^-2^ (± SD)*** | 23.89 (± 4.98) | 24.27 (± 5.28) | 0.61 |
| ***Sex ratio Male/Female (% women)*** | 0.36 (73.57) | 0.27 (78.60) | 0.31 |
| ***TSS, mean score (± SD)*** | 17.68 (± 3.80) | 19.19 (± 3.54) | 0.0002 |
| ***Mean duration of FD symptoms, months (± SD)*** | 67.36 (± 101.02) | 81.87 (± 98.76) | 0.18 |
| ***FD subtypes:***  ***PDS, n (%)***  ***EPS, n (%)***  ***PDS+EPS, n (%)*** | 88 (62.86)  20 (14.29)  32 (22.86) | 139 (64.65)  21 (9.77)  55 (25.58) | 0.74  0.23  0.34 |
| ***GIQLI, mean score (± SD)*** | 87.21 (± 16.88) | 72.62 (± 18.20) | < 0.0001 |
| ***GIQLI domains:***  ***symptoms, mean score (± SD)***  ***physical function, mean score (± SD)***  ***emotional function, mean score (± SD)***  ***social function, mean score (± SD)***  ***treatment, mean score (± SD)*** | 47.15 (± 7.95)  14.44 (± 5.50)  11.33 (± 4.09)  10.91 (± 3.71)  3.38 (± 1.06) | 42.61 (± 8.83)  9.43 (± 5.34)  9.14 (± 4.25)  8.36 (± 4.04)  3.07 (± 1.24) | < 0.0001  < 0.0001  < 0.0001  < 0.0001  0.023 |
| ***Smoking, n (%)*** | 18 (12.86) | 42 (19.53) | 0.11 |
| ***HADS, mean score (± SD)*** | 13.41 (± 6.14) | 18.66 (± 7.14) | < 0.0001 |
| ***HADS-A, mean subscale (± SD)*** | 8.40 (± 3.77) | 10.93 (± 4.23) | < 0.0001 |
| ***HADS-D, mean subscale (± SD)*** | 5.01 (± 3.44) | 7.73 (± 4.10) | < 0.0001 |
| ***Global PSQI, mean score (± SD)*** | 6.01 (± 2.45) | 10.82 (± 3.31) | < 0.0001 |
| ***Poor sleep quality (PSQI > 5), n (%)*** | 98 (70.00) | 214 (99.53) | < 0.0001 |
| ***ISI, mean score (± SD)*** | 5.26 (± 2.62) | 15.89 (± 4.16) | < 0.0001 |
| ***Insomnia (ISI ≥ 10), n (%)*** | 0 (0) | 215 (100) | < 0.0001 |
| ***IBS, n (%)*** | 55 (39.29) | 104 (48.37) | 0.10 |
| ***Heartburn, n (%)*** | 33 (23.57) | 63 (29.30) | 0.27 |
| ***Regurgitation, n (%)*** | 58 (41.43) | 99 (46.05) | 0.44 |
| ***Chest pain, n (%)*** | 26 (18.57) | 62 (28.84) | 0.033 |
| ***Any reflux symptom, n (%)*** | 69 (49.29) | 112 (52.09) | 0.66 |
| ***Gastric half-emptying time, minutes (± SD)**** | 163.84 (± 45.96) | 164.92 (± 69.59) | 0.64 |
| ***Delayed gastric emptying, n (%)**** | 17 (43.59) | 14 (35.90) | 0.64 |
| **BMI: Body Mass Index, EPS: Epigastric Pain Syndrome, FD: Functional dyspepsia, GIQLI: GastroIntestinal Quality of Life Index, HADS: Hospital Anxiety Depression Scale, HADS-A and HADS-D: HADS Anxiety and Depression subscales respectively, IBS: Irritable Bowel Syndrome, ISI: Insomnia Severity Index, n: number, PDS: Postprandial Distress Syndrome, PSQI: Pittsburgh Sleep Quality Index, SD: Standard Deviation, TSS: Total Symptom Score**  *** Only available for a subgroup of 78 (21.97%) patients.** | | | |
